# Immunopathological mechanisms in the early stage of *Mycobacterium avium* subsp. *paratuberculosis* infection via different administration routes in a murine model

**DOI:** 10.1371/journal.pone.0281880

**Published:** 2023-02-16

**Authors:** Jun Ho Lee, Hong-Tae Park, Soojin Shim, Suji Kim, Sang-Ho Woo, Dae-Yong Kim, Han Sang Yoo

**Affiliations:** 1 Department of Infectious Disease, College of Veterinary Medicine, Seoul National University, Seoul, Republic of Korea; 2 BK21 FOUR Future Veterinary Medicine Leading Education and Research Center, College of Veterinary Medicine, Seoul National University, Seoul, Republic of Korea; 3 Research Institute for Veterinary Science, College of Veterinary Medicine, Seoul National University, Seoul, Republic of Korea; 4 Department of Veterinary Pathology, College of Veterinary Medicine, Seoul National University, Seoul, Republic of Korea; Rutgers Biomedical and Health Sciences, UNITED STATES

## Abstract

*Mycobacterium avium* subspecies *paratuberculosis* (MAP) is the causative agent of Johne’s disease, a chronic emaciating disease of ruminants that causes enormous economic losses to the bovine industry, globally. However, there are still remaining clues to be solved in the pathogenesis and diagnosis of the disease. Therefore, an *in vivo* murine experimental model was tried to understand responses in early stage of MAP infection by oral and intraperitoneal (IP) routes. In the MAP infection size, and weight of spleen and liver were increased in the IP group compared with oral groups. Severe histopathological changes were also observed in the spleen and liver of IP infected mice at 12 weeks post-infection (PI). Acid-fast bacterial burden in the organs was closely related to histopathological lesions. In the cytokine production from splenocytes of MAP-infected mice, higher amounts of in TNF-α, IL-10, and IFN-γ were produced at early stage of IP-infected mice while IL-17 production was different at time and infected groups. This phenomenon may indicate the immune shift from Th1 to Th17 through the time course of MAP infection. Systemic and local responses in the MAP-infection were analyzed by using transcriptomic analysis in the spleens and mesenteric lymph nodes (MLN). Based on the analysis of biological processes at 6 weeks PI in spleen and MLN in each infection group, canonical pathways were analyzed with ingenuity pathway analysis in the immune responses and metabolism especially lipid metabolism. Infected host cells with MAP increased in the production of proinflammatory cytokines and reduced the availability of glucose at early stage of infection (*p* < 0.05). Also, host cells secreted cholesterol through cholesterol efflux to disturb energy source of MAP. These results reveal immunopathological and metabolic responses in the early stage of MAP infection through the development of a murine model.

## Introduction

Paratuberculosis or Johne’s disease (JD) caused by *Mycobacterium avium* subsp. *paratuberculosis* (MAP) is a chronic, untreatable and debilitating disease mainly concerning ruminants [1, 2]. The characteristic clinical symptoms are chronic diarrhea and progressive weight loss, and eventually animal death. Due to the long incubation period of MAP infection, the disease can spread to other susceptible animals as the bacteria are excreted in feces during the subclinical stage [3–5]. It has been reported that animals such as primates, rabbits, stoats, foxes, and weasels, including domestic and grazing ruminants, are susceptible to MAP [6–8], and a link has been suggested with human inflammatory disease, more precisely Crohn’s disease, a type of chronic inflammatory bowel disease called inflammatory syndrome [9]. JD has tremendous economic importance in the global bovine industry [3]. The disease has been received alteration as a result of a negative relationship with humans caused by Crohn’s disease, as well as a massive economic loss to the bovine industry [10]. This interest has resulted in an increasing number of studies examining host‒pathogen interactions related to MAP, and cattle are often used as natural hosts of MAP [11]. However, using ruminants as a clinical model for JD progression is time-consuming because the animal must be maintained until signs of disease develop [12, 13]. Additionally, ruminants are expensive and difficult to handle. A bovine model that enables the study of all stages that can occur through MAP infection has not been reported thus far. But several animal species, such as sheep, goats, and calves, have been used to study vaccine strains as the MAP infection model [14].

The development of a murine model for MAP studies is required for the early screening of vaccine candidates and preliminary analysis of pathogenesis [13]. However, typical features of JD, such as diarrhea and severe intestinal lesions occurring in cattle, cannot be reproduced in mice, and there are limitations in understanding granuloma development and mycobacterial latency to mycobacterial infection through a murine model [13, 15, 16]. However, the availability of many well-developed immunological reagents and mice of various genetic backgrounds make the murine model suitable for studying the mechanisms of MAP pathogenicity [13, 17, 18]. Histological and immunological features similar to those found in ruminants related to MAP infection were reproduced in mice [13, 17]. In a previous study comparing the various routes of infection in a murine MAP model, IP injection could reproduce the infection in all inoculated mice, whereas inoculation via the oral route induced MAP in only 58% of mice [13, 19]. This shows that IP injection has been frequently used for MAP infection in mice. The mouse strain is used differently depending on the number of lesions and the degree of bacterial colonization [13]. In this case, the BALB/c and C57BL/6 strains are susceptible to infection, and the C3H strain is more resistant [18, 20]. Traditionally, *Mycobacteria* use several host immune evasion strategies, such as preventing T-cell recognition of infected macrophages and evading macrophage-mediated killing by blocking phagosome acidification and maturation within the macrophage [21–23]. This is why the host immune response to mycobacterial infection is studied *in vitro* using macrophages.

Previously, analysis of host immune response and immunological characteristics for mycobacterial infection has been performed in animal models. One study, immunity response and mechanism of pathogenesis to MAP were observed in calves through ileal cannulation surgery [24]. Another study also observed the effectiveness of MAP infection by inoculating calves with MAP by oral and IP routes [25]. In addition, the level of infection and lesion development according to the MAP strain were compared [25]. These studies have been performed to reproduce MAP infections through different infection routes in ruminant animals, especially cattle, known as the main host of MAP. However, the mechanism and immune response observation of mycobacterial early infection based on two different administration routes (oral and IP route) by using murine model have not been studied. In one study, the MAP-related oral infection model was tested another study also by inoculating the murine oral model with MAP to observe immune response and pathology [26]. In another study, an examination of the host response to MAP infection in mice was performed by using the spleen through IP route [11]. We observed immunopathological responses in the early stages of MAP infection by comparing histopathological and immunological characteristics through a murine model infected with MAP by different administration routes. Comparisons of different administration routes can provide an understanding of the immunopathological response and the development of *in vivo* murine experimental models in early stage of MAP infection. In addition, transcriptomic analysis was performed using the spleen and MLN of the MAP-infected murine model. Immune responses and gene expression patterns according to host-pathogen interactions occurring in early stage of MAP infection were observed. It provides information on the mechanism of infection and the immune response between host-pathogen in the early stage of MAP infection according to two administration routes.

To date, various studies have been conducted to analyze the pathogenicity of MAP, but an accurate interpretation of the mechanism of early stage of MAP infection has not yet been established. Clinical signs of disease following MAP infection may not occur for a long time after infection, and this makes it difficult to clear the pathogen from the host. Understanding the pathogen‒host interaction is essential to control infection. Also, in order to identify potential control strategies, it also needs to develop an efficient animal model that mimics natural infection. Based on the observation of MAP infection-specific features, such as granuloma formation, in previous murine studies [11], the mouse is considered a promising MAP infection model. We evaluated the efficacy of MAP according to its ability to colonize tissues after oral and IP infection. Oral represents the natural route of infection, and IP is an effective route to induce infection in mice. Additionally, infection-specific changes, such as metabolic changes that may occur in the early stage of infection, overall gene expression patterns in immune cells, and bacterial shedding, were observed. By observing the mechanism of MAP infection in the latent or early stage of infection through this animal experiment, basic data for the development of diagnostic and prevention methods for JD might be provided.

## Materials and methods

### Ethics statement

All animal experiments were performed according to the principles of the Animal Protection Act and the Laboratory Animal Act in the Republic of Korea. Procedures for laboratory animals used in the experiments were conducted according to the guidelines and recommendations of the Institutional Animal Care and Use Committee (IACUC) of the Animal and Plant Quarantine Agency, Republic of Korea, and the protocol was reviewed and approved by the Seoul National University Institutional Animal Care and Use Committee (SNUIACUC) (permit no. SNU-190621-4).

### Preparation of bacterial strain

The *Mycobacterium avium* subsp. *paratuberculosis* ATCC 19698 strain was grown at 37°C on Middlebrook 7H10 agar medium (19 g, BD Biosciences, Sparks, MD, USA) containing 0.5% glycerol, Mycobactin J (2 mg/L, Allied Monitor, Fayette, MO), and 10% oleic-albumin-dextrose-catalase (OADC) enrichment (Beckton Dickinson, Sparks, MD, USA). Animal infections were performed by suspending MAP ATCC 19698 in phosphate-buffered saline (PBS) and diluting the cells to 1 × 10^9^ cells/mL.

### Animal infection

Five-week-old C57BL/6 female mice (OrientBio Co., Ltd., Republic of Korea) fed standard food and water *ad libitum* were used as the animal infection model and housed in polycarbonate cages (6 mice per cage) with proper temperature and humidity (22°C and 50% humidity). Animals were cared for in accordance with standard regulations and policies for the care and use of laboratory animals at Laboratory Animal Center in Seoul National University, Republic of Korea. Animal caretakers check mice in a 24-hour period. Since the bacterial strain used in the experiment is a microorganism that induces chronic infection, euthanasia was humanely performed as soon as possible with no pain after inhalation anesthesia with ether if it did not meet the criteria used as an endpoint in the chronic infection model. To perform animal infections, mice were inoculated with 1 × 10^9^ CFU of MAP ATCC 19698 by the oral and IP routes (MAP-challenged group), and mice inoculated with 300 μL PBS (control group) were also included. Six mice from each group were designed time scheduled euthanized by a CO_**2**_ chamber at 6, 12, and 18 weeks PI without unintended dead individuals. The size and weight of the spleen and liver of each group were measured and collected for histopathological evaluation. The murine model used in the experiment is not a natural host of the MAP, it does not cause a typical disease. Through this, the IACUC approval for the experiment was obtained.

### Hematoxylin and eosin staining

At each infection time point, four to five sections were extracted from the liver and spleen of each mouse, and tissue fixation was performed with 10% phosphate-buffered formalin. For general examination of the sample by the histopathological method, formalin-fixed tissue was routinely processed, deparaffinized, and stained with hematoxylin and eosin (H&E). Ziehl-Neelsen’s acid-fast stain was also used to stain another section of liver and spleen. In H&E staining, the lesion severity was scored from 0, when no lesion was present, to +1 to +5 when it was present. In detail, when a lesion was present, the frequency and size of granuloma observed in each tissue sample in the liver were graded from +1 to +5. The spleen was scored from +1 to +5 according to the degree of disappearance of the structures of white pulp and red pulp and the degree of penetration of macrophages.

### Acid-fast staining

Acid-fast staining was performed by randomly identifying 7 granulomatous lesions where acid-fast bacilli were observed in the liver and counting the number of bacteria therein. According to the number of bacteria, 0 if no bacteria were present, +1 to +5 if bacteria were present, the average was obtained (+1, 1–4; +2, 5–8; +3, 9–12; +4, 13–16; +5, 17>). For acid-fast staining on the spleen, 5 locations with lesions were selected and enlarged to count the number of bacteria in the 400-fold field. According to the number of bacteria, 0 if no bacteria were present, +1 to +5 if bacteria were present, the average was obtained (+1, 1–10; +2, 11–20; +3, 21–30; +4, 31–40; +5, 41>). The mean lesion scores of the liver and spleen at 6, 12 and 18 weeks PI were statistically compared by Student’s *t test*. Data are expressed as the means ± standard error (SE), and statistical significance was analyzed via ANOVA with Tukey’s multiple comparisons test.

### Detection of bacterial DNA with quantitative PCR

The extraction of total DNA from each sample (feces, small intestine, mesenteric lymph node, liver and spleen) of laboratory mice was performed using the AllPrep DNA/RNA Mini kit (Qiagen, Valencia, CA, USA) according to the manufacturers’ instructions. First, a bead beating step was performed to destroy the MAP cell wall. Briefly, lysis buffer (RLT) was added to the cells and applied to a 2 mL screwcap tube including 0.1 mm silica/zirconia beads. Then, bead beating was conducted twice using a FastPrep-24 5G instrument (MP Biomedicals, CA, USA) for 45 s at 6.5 speed. The lysate was centrifuged at 13,000×g for 2 min, and the supernatant was applied to the next step. Quality control of the extracted DNA was performed via measuring A260/230 and A260/280 ratios using an ND–1000 Spectrophotometer (NanoDrop, Waltham, MA, USA), and DNA purity and integrity were assessed. MAP DNA in each tissue sample was then measured via quantitative PCR using an IS900 gene as previously described [27].

### Measurement of cytokine production

To evaluate cytokines production upon stimulation with 10 μg/mL of MAP lysate, which was sonicated and quantified by the BCA assay, splenocytes from each group of mice (infected via the oral and IP routes) 6, 12 and 18 weeks PI with the MAP ATCC 19698 strain plus the control group (non-infected group mice) were collected. Cytokine production from the splenocytes of each group were measured by stimulation of splenocytes with PBS or MAP lysate. Briefly, 1 × 10^6^ cells/well were seeded into a 6-well plate and stimulated with MAP lysate for 12 h and 24 h at 37°C in 5% CO₂. Then, the culture supernatants were collected, and the quantities of TNF-α, IL-10, IFN-γ and IL-17 were measured by enzyme-linked immunosorbent assay (ELISA) using a mouse ELISA kit (Invitrogen) according to the manufacturer’s protocol.

### RNA-seq analysis

Total RNA was extracted from the mesenteric lymph node (MLN) and spleen of 6-week PI mice using an AllPrep DNA/RNA Mini kit (Qiagen, Valencia, CA, USA) at all three-time points following inoculation with the MAP ATCC 19698 by the IP and oral routes. The quality of extracted RNA was determined using A260/230 and A260/280 ratios with an ND–1000 Spectrophotometer (NanoDrop, Waltham, MA, USA) to assess RNA purity and integrity. Subsequent RNA preparation was performed by TeragenEtex Bio Institute (Seoul, Republic of Korea). Sequencing libraries were generated through the TruSeq Stranded mRNA Sample Preparation Kit (Illumina, CA, USA), and sequencing was performed in a paired-end form on the Illumina NovaSeq 6000 sequencer. After filtration of low-quality reads, the reads were mapped to the reference genome related to the species through Bowtie2 (version 2.3.5). Differentially expressed gene (DEG) analysis was performed with Cuffdiff (ver. 2.2.1), one of Cuffinks’ accessory tools [28], using the gene annotation database of the species. Gene Ontology (http://geneontology.org) and PANTHER Classification system (http://pantherdb.org/about.jsp) [29] were used to perform functional classification and analysis of genes whose expression changed.

### Biological system analysis

DEGs of both groups (IP and oral from MLN and spleen) were obtained compared to uninfected controls based on fold-change ≥ |2.0| and *p* value < 0.05. Analysis was performed by Ingenuity Pathway Analysis (IPA; Qiagen Inc., Hilden, Germany, https://www.qiagenbioinformatics.com/products/ingenuity-pathway-analysis) for canonical pathway and functional analyses [30]. DEGs with adjusted fold change ≥ |2.0| and *p* value < 0.05 were uploaded using the IPA program. Each gene was mapped to its corresponding gene acquisition using the Ingenuity Knowledge Base. Biological function analysis was performed using IPA to compare the DEGs related to disease and disorders, molecular and cellular functions, and physiological system development and function in mice infected with MAP by each route. Canonical pathways out of the IPA library of canonical pathways were examined to identify major biological pathways related to MAP infection in mice. The significance of the association between the canonical pathway and the dataset was affected by the ratio of the number of genes in the dataset mapped to a pathway to the total number of genes mapped to the canonical pathway.

### Statistical analysis

The statistical significance of internalization was analyzed by Student’s t test or repeated-measures ANOVA using GraphPad Prism software version 9.4.0 (GraphPad Software, San Diego, CA, USA, https://www.graphpad.com) and the data are represented as the means ± SE. Statistical significance was considered when the *p* value of each test was less than 0.05. All experiments were performed and recorded in triplicate.

## Results

### Comparison of histopathologic characteristics in different administration routes

**Fig 1:**
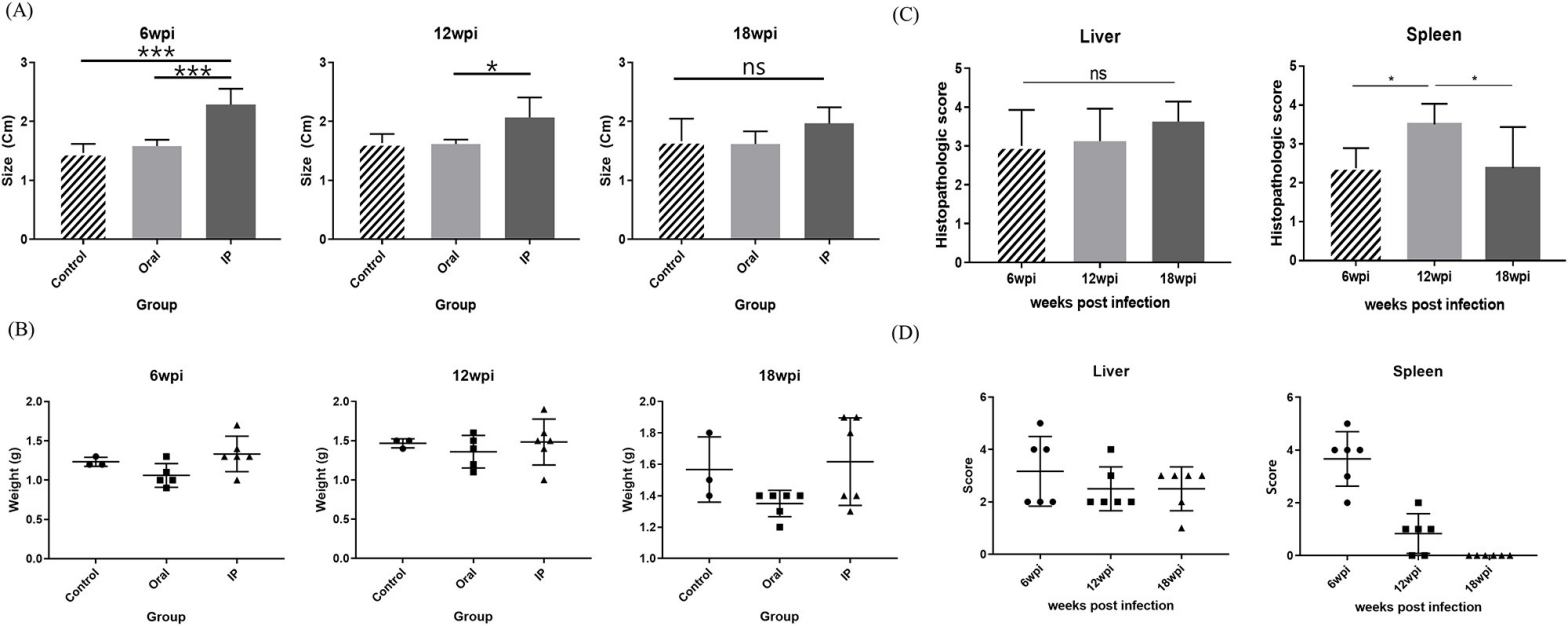
Histopathological characteristics in MAP-infected mice. (A) Changes in the size of the spleen according to the infection period in MAP-infected mice. (B) Changes in the weight of the liver according to the infection period in MAP-infected mice. (C) Histopathologic scoring after H&E staining. (D) MAP infection degree by acid-fast staining. Statistical significance was calculated by ANOVA with Tukey’s multiple comparisons test (*p* value, *<0.05; **<0.01; ***<0.0005; ****<0.0001).

Changes in histopathologic characteristics were observed in the liver and spleen of MAP-infected mice according to different administration routes during the experimental period. It was observed that the spleen enlarged throughout the entire period after IP infection while no specific changes were observed in the oral route (Fig 1A). The size of the spleen of mice infected through IP route was significantly larger than that of the oral route infected mice at 6 and 12weeks PI (Fig 1A). The liver weights of the IP group mice were higher than those of the oral group mice at 6, 12, and 18 weeks PI (Fig 1B).

During the IP infection period, where the size and weight of the spleen and liver were significantly higher than those in the oral group, histopathological evaluation through H&E staining and infection level evaluation through acid-fast staining were performed (Fig 1C and 1D). First, when the changing pattern of liver and spleen lesions in the IP group based on infection period was evaluated using histopathologic scoring of H&E staining, the degree of lesions in the liver tended to worsen over time even though there was significant difference. In the spleen, the histopathologic score was significantly higher at 12 weeks PI (*p* < 0.05), showing the most severe lesions, but no significant differences were observed at 6 or 18 weeks PI (Fig 1C). Next, when acid-fast staining of the liver and spleen samples of the IP group were scored according to the extent of bacterial distribution, there was large variation between individuals. In the case of the liver and spleen, the number of MAP bacteria gradually decreased with time, and the highest number of bacteria was observed on average at 6 weeks PI (Fig 1D).

MAP DNA was detected in 5 tissues (feces, mesenteric lymph nodes, small intestine, liver, and spleen) through quantitative PCR (q-PCR) analysis (Table 1). Thirty-five threshold cycle (Ct) values were set as cutoff standards to determine positive and negative values. When MAP detection in the oral group and IP group was compared, significant amounts of MAP were detected in the IP group, whereas little MAP were detected in the oral group during the infection period. In particular, in the IP group, a large number of MAPs were identified in the liver and spleen over the entire period, and MAP DNA was also detected in the small intestine and MLN. It was also observed that a small number of bacteria were shed with feces. In the oral group, bacteria were detected in the MLN, small intestine, and feces at 6 weeks PI, but the number was small, and there was a difference in the degree of detection for each individual. In addition, only small amounts of bacteria were detected in the liver and not at all in the spleen. However, in the case of mice infected by the oral route at 12 weeks PI, a small number of bacteria were detected in the MLN and spleen. At 18 weeks PI, no bacteria were detected in the liver or spleen, but a small amount was detected in the MLN. To detect bacteria from total DNA for each tissue sample, real-time PCR was performed on the IS900 gene.

**Table 1:** Detection of bacterial DNA in different organs of mice infected with MAP by q-PCR analysis.

		Feces	Mesenteric lymph node	Small intestine	Liver	Spleen
**Oral**	**6w**	2/5^a^ (31.2)^b^	4/5 (32.7)	1/5 (30.6)	1/5 (34.0)	-
	**12w**	-	2/6 (33.25)	-	-	2/6 (34.2)
	**18w**	-	1/6 (33.9)	1/6 (34.8)	-	-
**IP**	**6w**	4/6 (33.5)	6/6 (27.3)	6/6 (30.5)	6/6 (24.3)	6/6 (25.3)
	**12w**	1/6 (34.8)	6/6 (27.0)	6/6 (30.9)	6/6 (25.2)	6/6 (26.1)
	**18w**	-	2/6 (31.7)	3/6 (34.1)	6/6 (31.5)	6/6 (29.7)

^a^ positive number of samples/total number of samples,

^b^ average of Ct value (35 was the optimal cutoff)

### Analysis of immunological characteristics according to administration routes

**Fig 2:**
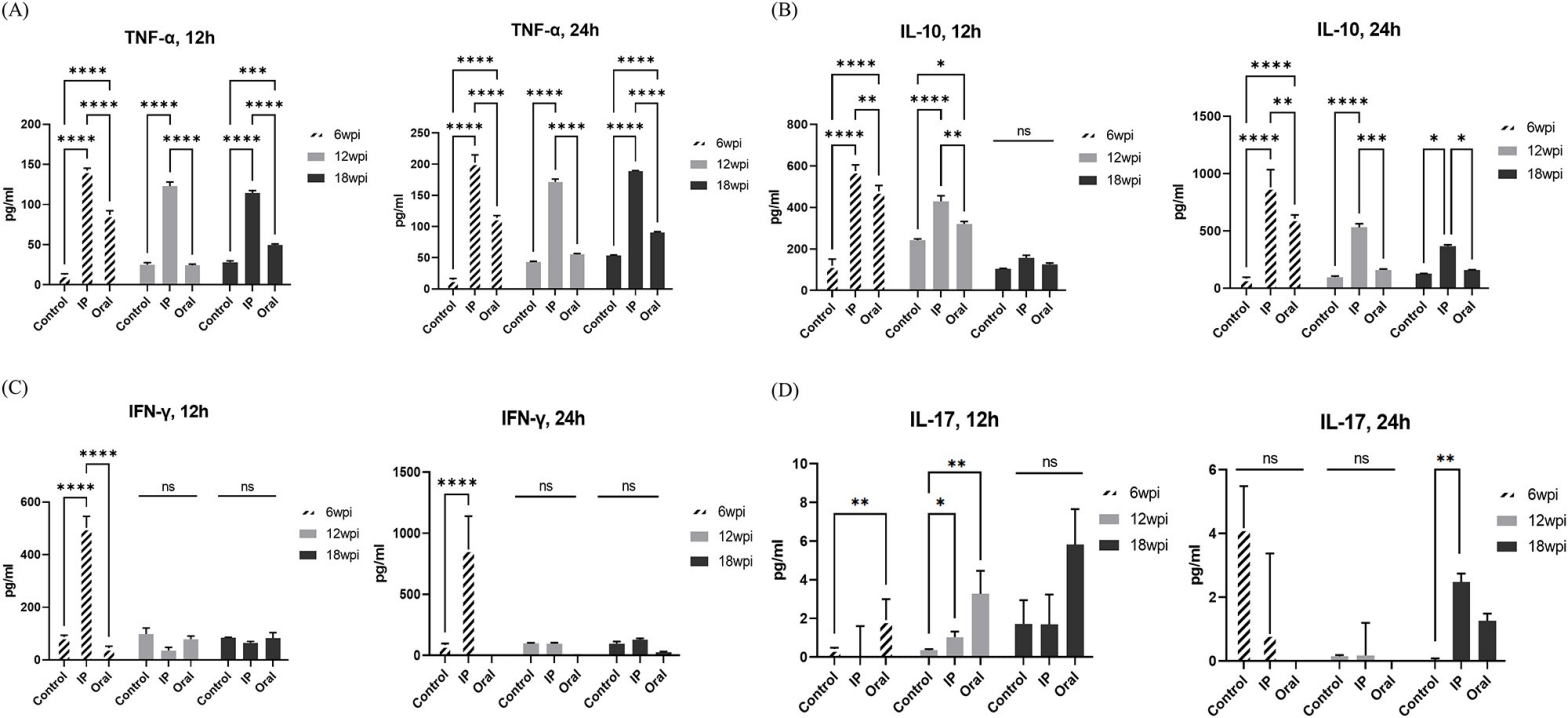
Immunological changes in the murine model after infection with MAP. Amounts of each cytokine in culture supernatant of splenocytes after stimulation with MAP lysates. (A) TNF-α. (B) IL-10. (C) IFN-γ. (D) IL-17. Statistical significance was calculated by ANOVA with Tukey’s multiple comparisons test (*p* value, *<0.05; **<0.01; ***<0.0005; ****<0.0001).

Splenocytes were used to observe various initial immune responses that may occur when MAP is infected through different administration routes (oral and IP routes). Amounts of cytokines (TNF-α, IL-10, IFN-γ and IL-17) were quantified from the supernatant by ELISA in response to stimulation with MAP cell lysate (Fig 2). TNF-α showed a significant increase in expression in both the oral and IP groups compared to the control group at 6 weeks PI and at 12 weeks PI, and only the IP group showed a significant increase in expression. At 18 weeks PI, a continuous increase in expression was observed in the IP group, and significantly higher expression was observed in the oral group than in the control group (Fig 2A). Significantly higher expression of IL-10 was observed in both the oral and IP groups than in the control group at 6 weeks PI and at 12 and 18 weeks PI, and significantly higher expression values were maintained in the IP group (Fig 2B). In the case of IFN-γ, significantly higher expression was observed in the IP group than in the other groups at 6 weeks PI, but no significant difference in expression was observed in any group at 12 and 18 weeks PI. After 24 hours of stimulation, the oral group showed lower expression values than the other groups in 12 and 18 weeks PI samples (Fig 2C). In the case of IL-17, significantly higher expression was observed in the oral group at 6 and 12 weeks PI than in the other groups, and the highest IL-17 production was induced at 12 hours after stimulation during the infection period. However, the overall expression level was low (Fig 2D).

### Analysis of differentially expressed genes by RNA-seq

**Fig 3:**
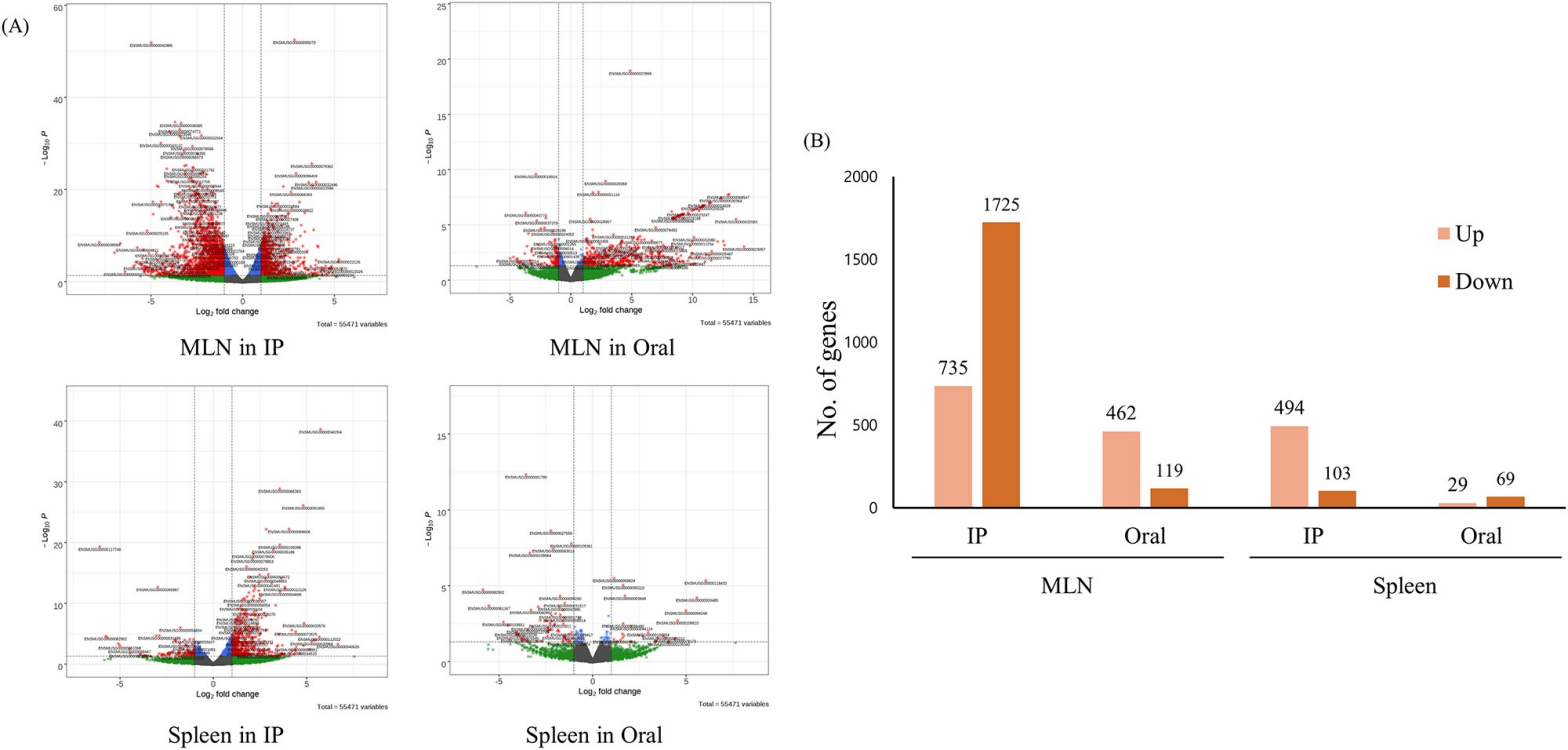
Comparison of gene expression levels between control and MAP-infected mice at 6 weeks PI according to different administration routes and organs. (A) Volcano plot analysis of DEGs in the MLN and spleen of the IP group and oral group. Red dots indicate an expression level change of fold change ≥ |2.0|, and green dots indicate a *p* value < 0.05. The expression levels were calculated using Cuffdiff from each sample. (B) The number of differentially expressed genes between control and MAP-infected mice according to different administration routes and organs.

DEG analysis was performed through RNA-seq data for gene expression analysis in the MLN and spleen according to infection routes of MAP-infected and control mice. DEG analysis compared the infection group (oral and IP route) and the non-infection control group in the MLN and spleen of 6-week PI mice, which showed the most significant histopathological and immunological characteristics according to different administration routes (fold change ≥ |2.0|, and *p* value < 0.05) (Fig 3A). Of the 55,471 analyzed genes, 2,460 (4.43%) in MLN of IP group, 581 (1.05%) in MLN of oral group, 597 (1.08%) in spleen of IP group and 98 (0.18%) in spleen of oral group genes were differentially expressed compared to the control group. Among them, 735 genes were upregulated and 1725 genes downregulated in the MLN of IP groups at the 6-week PI. In the MLN of the oral group, 462 genes were upregulated, and 119 genes were downregulated. In the spleen of the IP group at 6 weeks PI, 494 genes were upregulated while 103 genes were downregulated. In the spleen of the oral group, 29 genes were upregulated, and 69 genes were downregulated (Fig 3B).

The top 10 up- and downregulated genes according to each pathway from the MLN and spleen of MAP-infected mice at 6 weeks PI are listed in S1 Table. Upregulated genes induced by MAP infection in the MLN were found to be related to immune response (*Olfm4*, *Acod1*, *Ighv9-4*, *Hpx*, *Fgg*, *Fgb*), signal transduction (*Acod1*, *Ighv9-4*, *Ms4a3*, *Hpx*, *Fgg*, *Fgb*), lipid metabolism (*Cyp3a11*, *Apob*), cell structure and motility (*Apob*, *Fgg*, *Fgb*) and other metabolic processes (*Mcpt8*, *Stfa2l1*, *Cyp3a11*, *Apob*, *Fgg*, *Fgb*). Upregulated genes induced by MAP infection in the spleen were found to be related to the immune response (*Saa3*, *Gbp2b*, *Ighv1-78*), signal transduction (*Gm42878*, *Ighv1-78*, *Gabrb1*), cell structure and motility (*Pdilt*, *Ighv1-78*) and other metabolic processes (*Gm42417*, *Gm42878*, *Gabrb1*). The most upregulated genes among them were related to immunity and metabolism. The most upregulated neutrophil granule protein (*Olfm4*) in the MLN of MAP-infected mice at 6 weeks PI plays an important role in innate immunity against bacterial infection, and the most upregulated acute phase protein (*Saa3*) in the spleen suggested that MAP-infected mice at 6 weeks PI at an early stage of MAP infection along with *Olfm4*.

In contrast to upregulated genes, downregulated genes induced by MAP infection in the MLN were found to be related to immune response (*Reg3a*, *Igkv3-3*), signal transduction (*Ins2*, *Faim2*), lipid metabolism (*Ins2*, *Dhrs9*), cell structure and motility (*Cldn22*, *Reg3a*, *Ins2*, *Tmed11*, *Faim2*) and other metabolic processes (*Amy2a5*, *Reg3a*, *Ins2*, *Tmed11*, *Faim2*, *Igkv3-3*, *Capn11*). In the spleen, it was found to be related to immune response (*Ighv1-37*, *Igkv4-72*), signal transduction (*Ighv1-37*), cell structure and motility (*Ighv1-37*, *Krt14*, *Lhx9*) and other metabolic processes (*Mcpt4*, *Cpne4*, *Ighv1-37*, *Gm45713*, *Lhx9*, *Igkv4-72*). The most downregulated genes were associated with signal transduction, cell structure and motility and metabolism.

### Analysis of gene ontology

**Fig 4:**
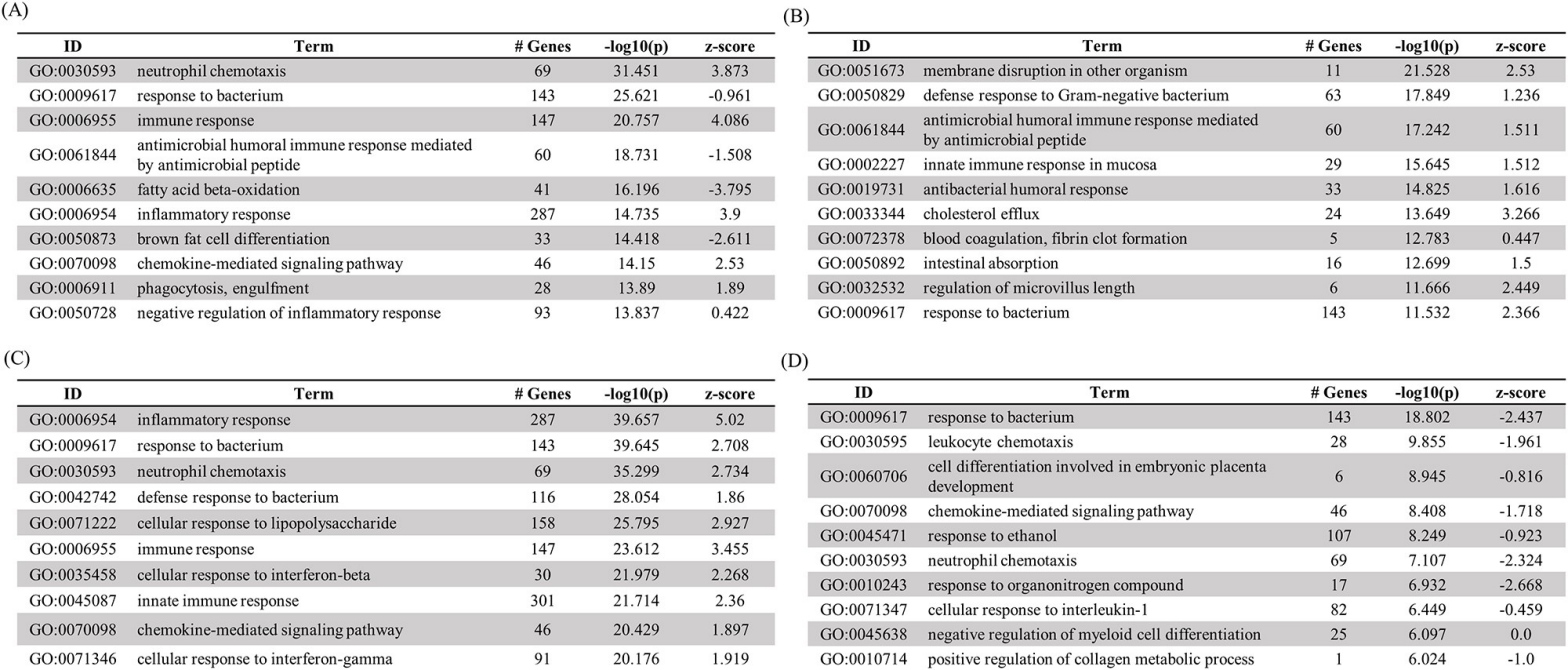
Top 10 Gene Ontology terms (biological process) of mice at 6 weeks of MAP infection. (A) MLN administered by the IP route, (B) MLN administered by the oral route, (C) spleen administered by the IP route, (D) spleen administered by the oral route.

Gene ontology (GO) analysis was performed to classify the functions of genes whose expression changed according to each group (oral and IP groups) from the MLN and spleen of 6-week PI mice. GO enrichment analysis of these genes was performed by PANTHER bioinformatics tools. The GO terms enriched in DEGs for each group were organized into three categories: biological process, cellular component, and molecular function. The top 10 GO terms in the biological process category are summarized in Fig 4. As a result of sorting and analyzing the terms of the biological process according to the *p* value, the terms related to the immune response were mainly mapped in the MLN of the IP group, and the genes related to lipid metabolism tended to suppress their expression (Fig 4A). In the MLN of the oral group, as in the IP group, terms related to the immune response were mainly mapped, and the term related to cholesterol efflux was also mapped (Fig 4B). In the spleen of the IP group, the term related to the inflammatory response was most significantly mapped, and the other top 10 terms were also related to the immune response (Fig 4C). In the spleen of the oral group, a relatively low *p* value was observed compared to other groups; unlike the IP group, a tendency toward suppressed expression of immune response-related genes was observed in both mapped GO terms (Fig 4D).

### Analysis of canonical pathway

For the analysis of gene expression according to different administration routes at the early stage of infection, the analysis focused on the canonical pathway targeting MAP-infected mice at 6 weeks PI, which showed the most significant histopathological and immunological characteristics from the spleen and MLN. As shown in S1 Table, the genes related to immune response and metabolism were most highly expressed in the MLN and spleen according to DEG analysis. As shown in Fig 4, many terms related to immune response were mapped through GO analysis, and cholesterol efflux-related terms were additionally mapped. These results made it possible to confirm the immune response induced by host cells against MAP and gene expression according to lipid metabolism through the canonical pathway. To perform canonical pathway analysis, three groups were divided according to the administration route and sample (spleen, MLN): MLN (IP route vs. oral route), spleen (IP route vs. oral route), and IP route (MLN vs. spleen). The IPA tool was used to perform canonical pathway analysis of three groups divided according to administration route (oral, IP) in the spleen and MLN. Among the significant pathways with *p* values < 0.05, the top 50 canonical pathways were sorted through comparison analysis to find pathways that were commonly activated by linkage to immune response and lipid metabolism (S1–S3 Figs). By comparing the z scores of the top 50 canonical pathways, the difference in the upregulated and downregulated levels of each pathway was analyzed.

### Comparison of gene expression according to administration routes in MAP-infected mice

As a result of canonical pathway alignment, immune response-related “TREM-1 Signaling”, “Role of Pattern Recognition Receptors in Recognition of Bacteria and Viruses”, “Th1 Pathway” and “Production of Nitric Oxide and Reactive Oxygen Species in Macrophages” pathways in the three groups showed upregulated levels according to z score (S1–S3 Figs). In addition, as a result of alignment of canonical pathways related to lipid metabolism, the “LXR/RXR Activation”, “Oxidative Phosphorylation” and “Fatty Acid β-oxidation I” pathways showed downregulation levels among the top 50 canonical pathways according to z score in the three groups (S1–S3 Figs).

**Fig 5:**
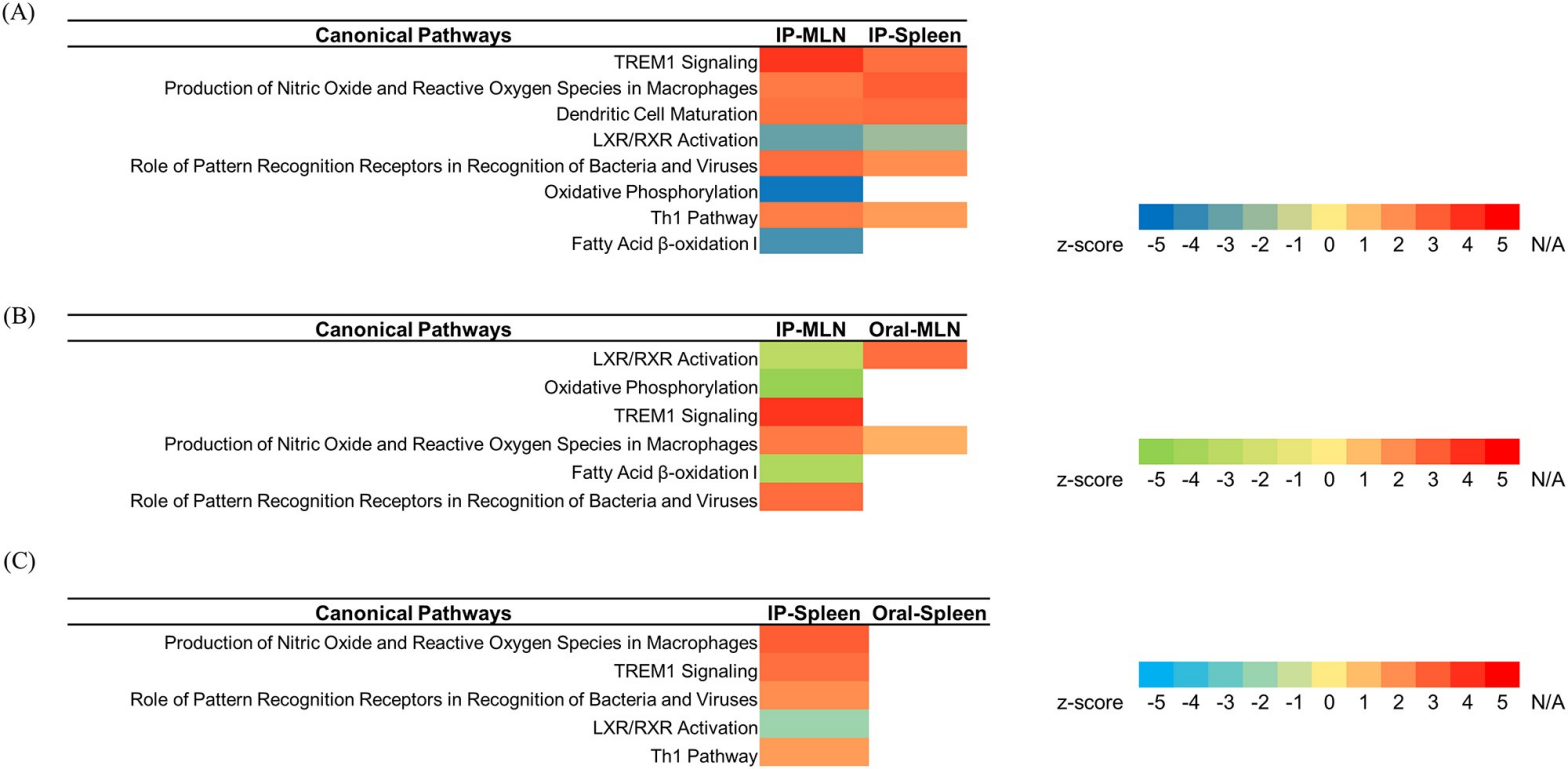
Canonical pathway analysis of DEGs related to immune response and lipid metabolism from each group using the IPA tool. Canonical pathways sorted by z score within the top 50 canonical pathways from the (A) IP route (MLN vs. spleen), (B) MLN (IP route vs. oral route), and (C) spleen (IP route vs. oral route). Genes that were not significant (*p* value ≥ 0.05 or Log_2_FC < 1.0) are shown as N/A.

Immune responses and lipid metabolism from the canonical pathway analysis are shown in Fig 5. In relation to the immune response, a comparison of the 6-week PI MLN (IP route vs. oral route) group showed that upregulation of the pathway was observed only when MAP was inoculated by the IP route, and the upregulation level was higher when compared with the oral route. In the spleen (IP route vs. oral route), upregulation of the immune response-related pathway occurred only when MAP was inoculated by the IP route, and the upregulation level was higher than that of the oral route. Therefore, through comparison of the IP and oral routes in the MLN and spleen, we found that the level of the pathway related to the immune response was further increased when MAP was inoculated by the IP route. When the IP route (MLN vs. spleen) group was analyzed based on comparison with the previous two groups, the upregulation level of the immune response-related pathway of the MLN was higher than that of the spleen. Finally, the upregulation of the immune response-related pathway was higher when MAP was inoculated through the IP route in the MLN (Fig 5A). As a result of canonical pathway analysis related to lipid metabolism, the downregulation level was found to be higher when MAP was inoculated by the IP route compared with the ’IP route vs. oral route’ group of MLN and spleen. Based on this, when applied to the IP route (MLN vs. spleen), the downregulation level of the pathway was higher when MAP was inoculated through the IP route to the MLN (Fig 5B). Overall, higher upregulation and downregulation levels were observed in the IP route than in the oral route for both canonical pathways related to immune response and lipid metabolism. Based on this, higher upregulation and downregulation levels were observed in the MLN than in the spleen when inoculated via the IP route with MLN and spleen.

**Fig 6:**
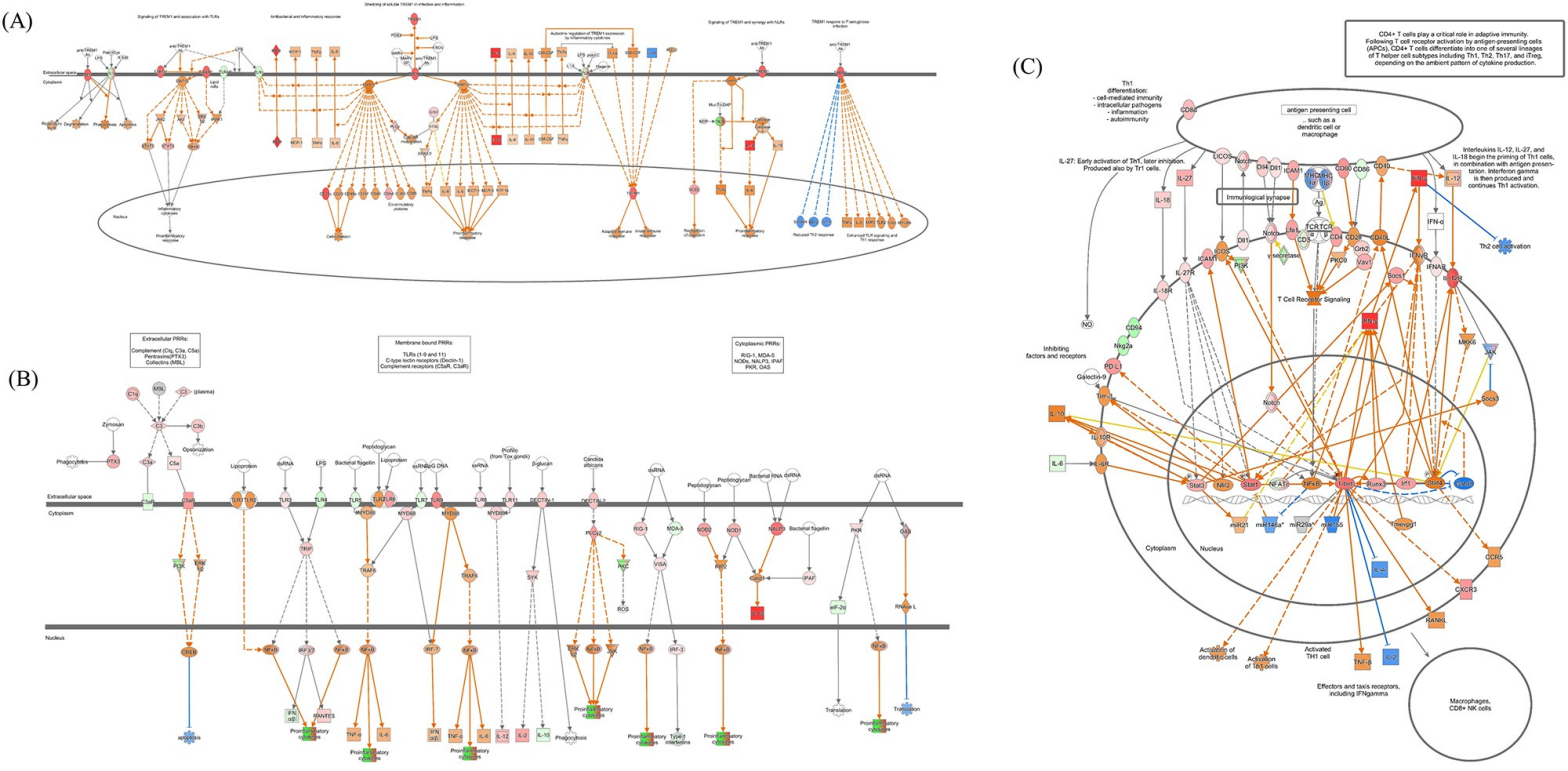
Ingenuity pathway analyses. TREM-1 signaling (A), Role of Pattern Recognition Receptors in Recognition of Bacteria and Viruses (B) and Th1 Pathway (C) in the MLN of the IP group at 6 weeks PI. Nodes are colored based on gene expression along with predictions of upstream and downstream molecules. A gene in red indicates upregulation, a gene in green indicates downregulation, a gene in orange indicates predicted activation, and a gene in blue indicates predicted inhibition. Uncolored nodes indicate that genes were not differentially expressed in the pathway shown.

**Fig 7:**
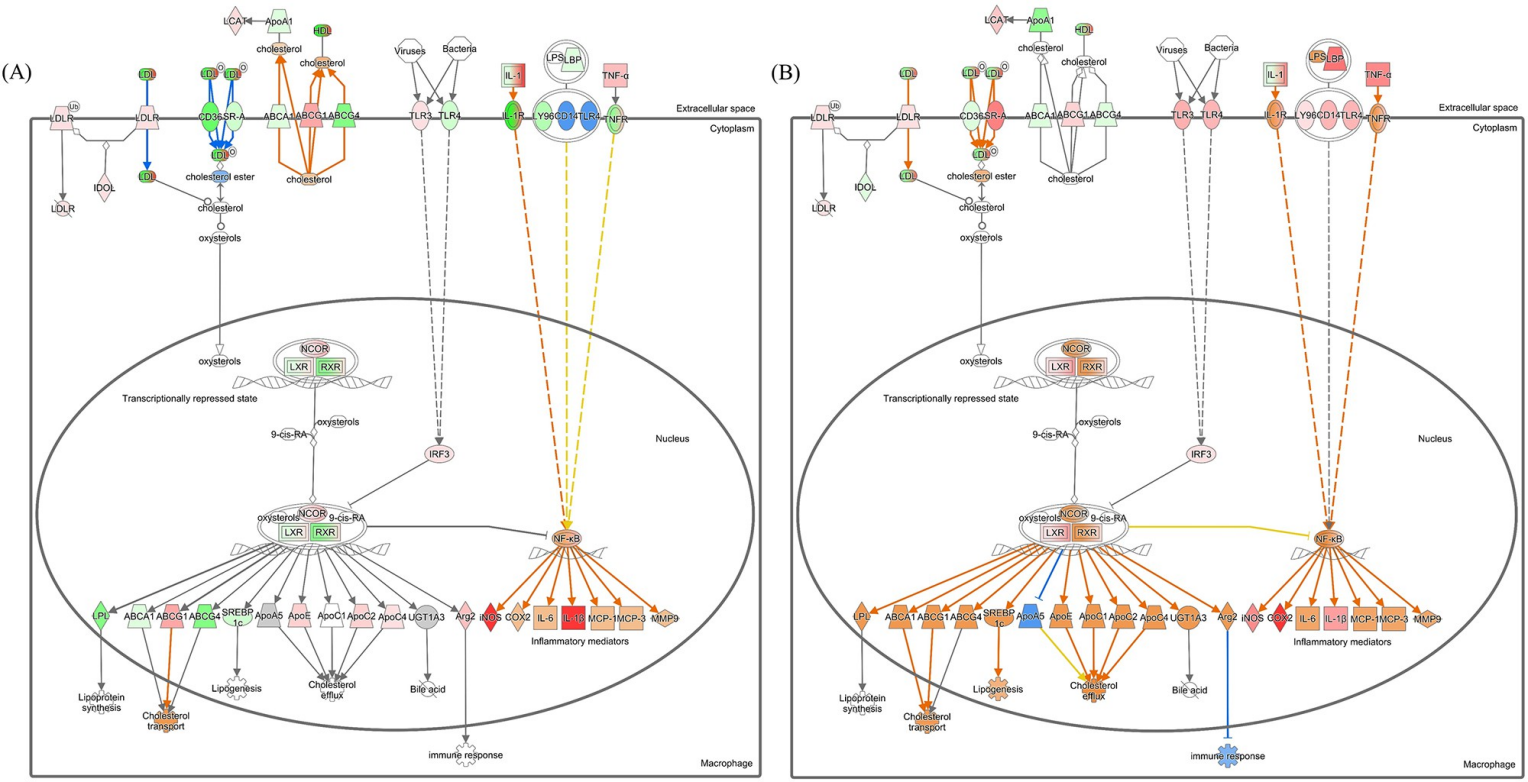
LXR/RXR activation pathways in the (A) MLN and (B) spleen of the IP group at 6 weeks PI. Nodes are colored based on gene expression along with predictions of upstream and downstream molecules. A gene in red indicates upregulation, a gene in green indicates downregulation, a gene in orange indicates predicted activation, and a gene in blue indicates predicted inhibition. Uncolored nodes indicate that genes were not differentially expressed in the pathway shown.

**Fig 8:**
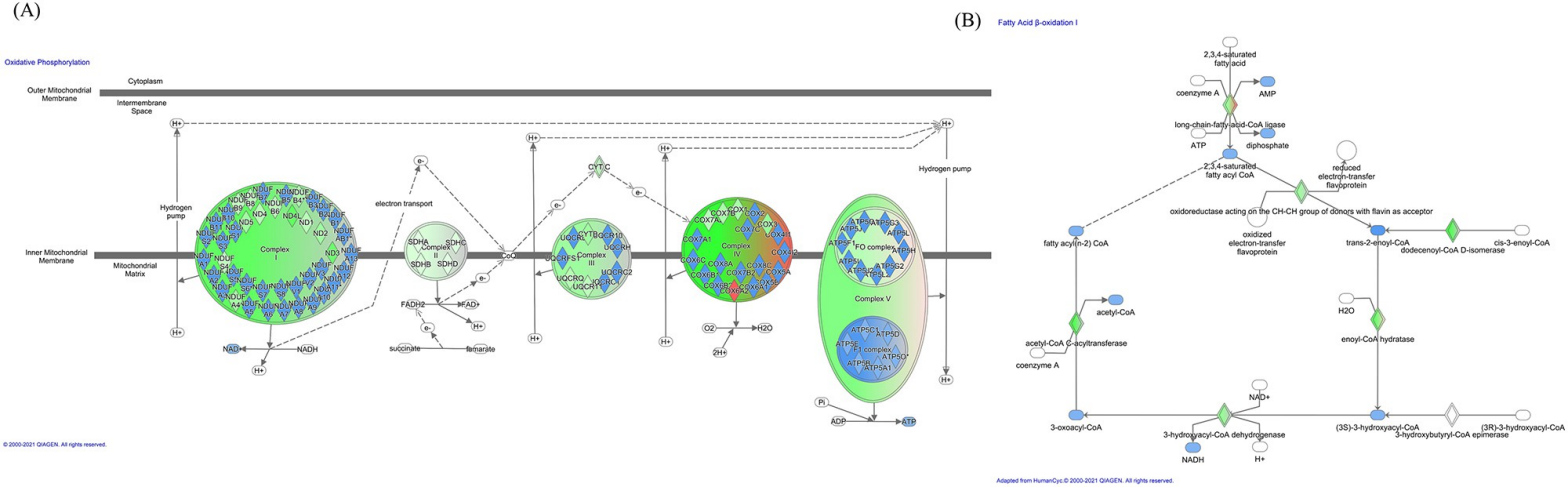
Ingenuity pathway analyses of oxidative phosphorylation (A) and fatty acid β-oxidation I (B) in the MLN of the IP group at 6 weeks PI. Nodes are colored based on gene expression along with predictions of upstream and downstream molecules. A gene in red indicates upregulation, a gene in green indicates downregulation, a gene in orange indicates predicted activation, and a gene in blue indicates predicted inhibition. Uncolored nodes indicate that genes were not differentially expressed in the pathway shown.

Overall, administration by the IP route during MAP infection and analysis of canonical pathways related to immune response and lipid metabolism in MLN were significant. As shown in Fig 6, host cell infection following MAP infiltration caused an increase in the secretion of proinflammatory cytokines, such as IFN-γ, TNF-α, and IL-1β, through the upregulation of pathways related to the immune response in the MLN of the IP group at 6 weeks PI. The predicted activation and expression of several genes involved in cholesterol transport and efflux, including genes encoding ATP-binding cassette, subfamily A, member 1 (ABCA1), ATP-binding cassette, subfamily G, member 1 (ABCG1), apolipoprotein C2 (ApoC2), and apolipoprotein E (ApoE), could be observed through the “LXR/RXR activation” pathway, which was common to the IP route (MLN vs. spleen) group (Fig 7). It was also observed that downregulation of ATP production processes through “Fatty acid β-oxidation” and “Oxidative phosphorylation” pathways blocked the use of ATP by MAP in host cells (Fig 8).

## Discussion

In the early stage of infection, other mycobacteria, including MAP, invade the host and preferentially infect macrophages, evading and redirecting the host’s immune response [4]. Bacteria are present in the phagosomes or early endosomes of host macrophages after crossing the M cells of ileal Peyer’s patches through transcytosis [31, 32]. MAP enables survival and proliferation in host macrophages through granuloma formation, and the survival of bacteria in macrophages is due to various mechanisms such as interfering with phagosome maturation or inhibiting immune-regulatory pathways [33, 34]. From the early stage of MAP infection to the early subclinical stage, the Th1-type immune response is dominant in relation to the host cell-mediated response, which plays an important role in the host’s antimycobacterial response against MAP [11, 32]. Then, with the progression of MAP infection, a temporal shift from the Th1 type to the Th2 type occurs when reaching the clinical stage, which is called a classical switch profile [32, 35]. The survival mechanism of MAP against the host in early infection makes it difficult to determine whether an individual is infected or not, and emphasizes the need to develop an initial diagnosis method for JD infection. However, the concept of the early stage of MAP infection mechanism has not yet been established. And it is also necessary to establish an experimental animal model for the development of the mycobacterial early infection diagnosis method. To date, analysis of histopathological and immunological characteristics and gene expression analysis related to early stage of MAP infection have not been performed in mice. Therefore, the *in vivo* murine model was established in early stage of MAP infection by infecting MAP through different administration routes and observing immunopathological response. And gene expression and immune response by using spleen and MLN related to systemic and local response in the early stages of MAP infection were observed. This study provides an understanding of the host‒pathogen relationship, infection-specific changes and mechanisms that may occur in the early stages of MAP infection using a murine model.

We analyzed the histopathological and immunological characteristics of the early stages of MAP infection via IP and oral route. And observed the gene expression using spleen and MLN in early stage of MAP infection through RNA-seq. The differences in changes between the two administration routes (oral, IP route) in relation to the early stage of MAP infection of the murine model are summarized in a table (Table 2). To analyze the histopathological characteristics in the MAP-infected and control groups in the murine model, the liver and spleen were extracted, and sampling was performed. The murine model inoculated by the IP route had an enlarged spleen and increased liver weight throughout the entire period after infection; in the murine model inoculated by the oral route, no specific changes were observed, or the changes were less than those of the IP route. The increase in the size and weight of the liver and spleen of MAP-infected mice was also found in MAP infection experiments of C57BL/6 mice by the IP route [36], and it was also found following MAP infection experiments of immunodeficient beige mice by the IP route [19]. In addition, granulomatous lesions characteristic of MAP infection were observed through H&E staining of liver and spleen tissues of control mice together with the experimental group inoculated both intraperitoneally and orally with MAP. No specific lesions were observed in the tissues of 18-week PI mice inoculated orally, but lesions were observed in the tissues of mice inoculated intraperitoneally at 6, 12, and 18 weeks PI. Finally, the observation of granulomatous lesions through H&E staining of liver and spleen tissues confirmed the observation of early lesions of MAP infection via the IP route, which were not observed via the oral route. This result was also found in a previous experiment in which granulomatous lesions were observed at 3 and 6 weeks PI at the initial stage of IP MAP infection in the liver and spleen using BALB/c mice [18, 37].

**Table 2:** Comparison of pathological, immunological and gene expression in mice infected oral or IP with MAP.

	Oral route		IP route	
**Histopathological changes**	Spleen	Liver	Spleen	Liver
	No specific changes were observed after infection	Little change in weight after infection	Enlarged throughout the entire period after infection	Increased compared to control and oral route after infection
**Cytokine production in splenocytes**	TNF-α: Increased cytokine production over all time periods IFN-γ: No significant cytokine production difference was observed IL-10: Significantly increased cytokine production in 6 weeks PI IL-17: Low overall cytokine production		TNF-α: Continuous increase in cytokine production throughout the entire period IFN-γ: Significantly increased cytokine production at 6 weeks PI IL-10: Significantly increased cytokine production over the entire period IL-17: Low overall cytokine production	
**Gene Ontology analysis**	Spleen	MLN	Spleen	MLN
	Suppressed expression of immune response-related genes	Mapping of terms related to immune response and cholesterol efflux	Mapping of terms related to inflammatory response and immune response	Mapping of terms related to immune response Suppressed expression of lipid metabolism related genes
**Canonical pathway analysis at 6 weeks PI**	IP vs Oral in spleen	Upregulation of the immune response related genes and downregulation of lipid metabolism-related genes in the IP group.		
	IP vs Oral in MLN			
	MLN vs spleen in IP	Upregulation of immune response related pathways in MLN. Downregulation of lipid metabolism related pathways in MLN.		

Ct value analysis was performed to confirm the distribution of bacteria according to the period after IP and oral inoculation in each tissue. In the case of the IP group, amplification of the IS900 gene was observed throughout the entire period except in feces at 18 weeks PI. In addition, a small number of bacteria were shed even in feces through the IP route. Based on these results, it is thought that inoculation of MAP through the IP route induces systemic infection. In the case of the oral group, bacteria were detected in all tissues except for the spleen at 6 weeks PI, but the number was small, and the degree of detection differed according to the period of infection. Thus, when MAP is inoculated through the oral route, local infection in the intestinal tract is maintained. However, in the case of mice orally inoculated at 12 weeks PI, although there are a small number of bacteria detected in the MLN and spleen, infection within the individual continues even in the absence of lesions.

Early immunological analysis of MAP-infected mice demonstrated host responses following 6, 12 and 18 weeks of infection, including activation of macrophages and MAP-killing activity. A significant increase in the expression of TNF-α was observed in both the oral and IP groups at 6 weeks PI, but a continuous increase in expression was observed in the IP group. TNF-α is a proinflammatory cytokine released after mycobacteria make initial contact with macrophages [38], and it activates naive macrophages and various T cells to prevent bacterial dissemination during the early stage of infection [4]. It was also reported to induce apoptosis of MAP-infected cells with antimicrobial effects [23]. Therefore, high expression of TNF is a mechanism to block the propagation of mycobacteria in the host, and in particular, the mycobacterial inhibition mechanism occurred significantly in the IP group. Similar to TNF-α, the production of IL-10 was also observed to be significantly high in both the oral and IP groups at 6 weeks PI. However, after that, a significantly high expression value was maintained only in the IP group. This is because MAP induces the expression of IL-10, an anti-inflammatory cytokine that inhibits macrophage cytokine release following MAP infection and improves bacterial intracellular growth [39]. Previous data from *Mycobacterium tuberculosis* (Mtb) studies demonstrated that IL-10 regulates the release of TNF-α, decreases the activity of TNF-α, and induces a decrease in apoptosis [23, 40]. MAP infection elicits an initial strong T helper 1 (Th1)-mediated response that is dominated by IFN-γ–secreting T cells [31]. However, as the disease progresses, the Th2 response becomes more prominent than the Th1 response, and the IFN-γ response is barely detected in the later stages of the disease [32]. Although the expression of IFN-γ was significantly higher than that of other groups at 6 weeks PI of the IP group, no significant difference in expression was observed across groups at 12 and 18 weeks PI and previous study supports this finding [32]. In the process of transitioning to the late stage of MAP infection from the early stage of MAP infection, the expression of TGF-β along with IL-10 is upregulated in the macrophages harboring high numbers of MAP [41]. An increase of TGF-β expression induces Th1 cells to express IL-17 and regulates Th1 converted into Th17 cells [42]. Thus, an increase in IL-17 expression over time in the oral group provides information on the immune shift from Th1 to Th17 via the time course of MAP infection. In addition, IL-17A is involved in the formation of granulomas by increasing chemokine production, which helps recruit inflammatory cells migrating to the infection site [43]. The peripheral blood of naturally MAP-infected animals induces higher levels of IL-17 than blood from healthy cows [44]. Therefore, when high levels of IL-17 were induced using a murine model of MAP infection, significantly higher expression was observed in the oral group at 6 and 12 weeks PI than in the other groups. In the two infection groups, the IP infection model appeared to replicate true infection (lesion, systemic localization of MAP, production of IFN-γ). However, regarding the response of IL-17 induced by MAP, further study of the oral infection model is required to understand the natural infection.

For pathogen infections, the spleen and MLN are appropriate organs to observe the response to systemic infection and local infection [45, 46]. Transcriptomic analysis was used to observe and analyze host-pathogen interactions in the spleen and MLN that are associated with systemic and local response of early stage of MAP infection. Regarding the immune response, the upregulation level of the IP group was higher than the oral group. In lipid metabolism, the downregulation level of the IP group was higher the oral group. Based on this, the spleen and MLN in the IP group were compared. In MLN, upregulation levels related to immune response and downregulation levels related to lipid metabolism were higher than in the spleen. It can be seen that the level of expression for immune response and lipid metabolism between host-pathogen interactions of early stage of MAP infections is higher in MLN related to local response.

MAP infects host cells and tries to maintain mycobacterial persistence. At this time, infection of host cells (such as macrophages) according to MAP infiltration increases the secretion of proinflammatory cytokines, such as IFN-γ, TNF-α, and IL-1, and induces hypoglycemia [47–49]; by reducing the availability of glucose in the host cell. This allows MAP to utilize cholesterol as an alternative energy source [50]. The use of cholesterol in host cells reduces the expression of MHC and costimulatory molecules (MHC, CD80, CD86) and reduces recognition by the immune system [51]. Unlike immune-sensitive cells, which have increased expression of costimulatory molecules by using glucose as an energy source, cells that evade the immune system by using lipids, such as cholesterol, as a primary energy source are classified as immune privileged [51]. In the end, the use of cholesterol changes the metabolic profile of the cell, resulting in an ’energy switch’ in which MAP uses cholesterol instead of glucose as an energy source, thereby avoiding the host immune system [50]. As mentioned above, this causes changes in cell metabolism and causes MAP-infected cells to evade the host immune system, resulting in mycobacterial persistence. As shown in Fig 6, when canonical pathway analysis was performed in the MLN and spleen of 6-week PI MAP-infected mice inoculated via the IP route, increased secretion of proinflammatory cytokines from host cells was observed. The increase in cytokines induces hypoglycemia in the early stage of MAP infection, and MAP induces the accumulation of cholesterol in the cell [50, 52]. That is, MAP uses cholesterol as a carbon source to generate ATP and obtains energy [50]. When MAP induces cholesterol accumulation for energy use in the host cell, it is thought that the host cell recognizes that there is too much cholesterol inside and exports it through cholesterol transport and efflux as a negative feedback mechanism (Fig 7). These results are consistent with previous studies that reported upregulation of ABCA1 and APOE, which are known as lipid efflux genes in the early stages of MAP infection, in macrophages infected with MAP strains [52]. It has been reported that upregulation of lipid efflux genes plays an important role in preventing intracellular cholesterol accumulation and inhibiting mycobacterial growth and survival [53, 54]. In this way, MAP also produces ATP through cholesterol, and it is thought to block the use of ATP by downregulating ATP production through β-oxidation, the TCA cycle, and oxidative phosphorylation in the host cell (Fig 8).

In this study, we compared and observed the immunopathological responses in the early stages of MAP infection according to oral and IP administration through histopathological and immunological characteristics. A comparison of the natural infection route of MAP (oral route) with the reproducible infection route in the murine model (IP route) can provide information on the development of *in vivo* murine experimental models that can observe the immunopathological response in early stage of MAP infection. In addition, transcriptomic analysis was used to confirm gene expression patterns using spleen and MLN representing systemic and local response. Through this, the relationship between host-pathogen in early stage of MAP infection was observed. These results allow the murine model to observe infection-specific changes like host-pathogen immune responses or gene expression patterns occurring in early stage of MAP infection. It also provides the immunological and metabolic responses against pathogens and the mechanism of early stage of MAP infection in the incubation period or sub-clinical state. As a result, we developed a reproducible murine model of MAP by different routes of infection, demonstrating immunopathological responses that were difficult to identify in real infected animals (cows), which may be useful for future investigations of disease pathogenesis and prevention. By observing histopathological and immunological characteristics and performing transcriptomic analysis, it is possible to provide information of immunopathological and metabolic responses according to host-pathogen interactions, including the mechanism of early stage of MAP infection.

## Supporting information

S1 Fig Canonical pathway analysis of DEGs from MLN and spleen in MAP infected mice by different administration routes using the IPA tool. Top 50 canonical pathways sorted by z score from the MLN and spleen in the IP route. Genes that were not significant (*p* value ≥ 0.05 or Log2FC < 1.0) are shown as N/A.
(TIF)

S2 Fig Canonical pathway analysis of DEGs from the MLN and spleen in MAP-infected mice by different administration routes using the IPA tool. Top 50 canonical pathways sorted by z score in the MLN by the IP and oral routes. Genes that were not significant (*p* value ≥ 0.05 or Log2FC < 1.0) are shown as N/A.
(TIF)

S3 Fig Canonical pathway analysis of DEGs from the MLN and spleen in MAP-infected mice by different administration routes using the IPA tool. Top 50 canonical pathways sorted by z score in the spleen by the IP and oral routes. Genes that were not significant (*p* value ≥ 0.05 or Log2FC < 1.0) are shown as N/A.
(TIF)

S1 Table The 10 most up- or downregulated genes in the MAP-infected mice (at 6 weeks mouse PI). (DOCX)
