# Supplementary figures and images for: Immunopathological mechanisms in the early stage of Mycobacterium avium subsp. paratuberculosis infection via different administration routes in a murine model
Source: PLoS One. 2023 Feb 16;18(2):e0281880. doi: 10.1371/journal.pone.0281880 (PMC9934400; doi:10.1371/journal.pone.0281880)

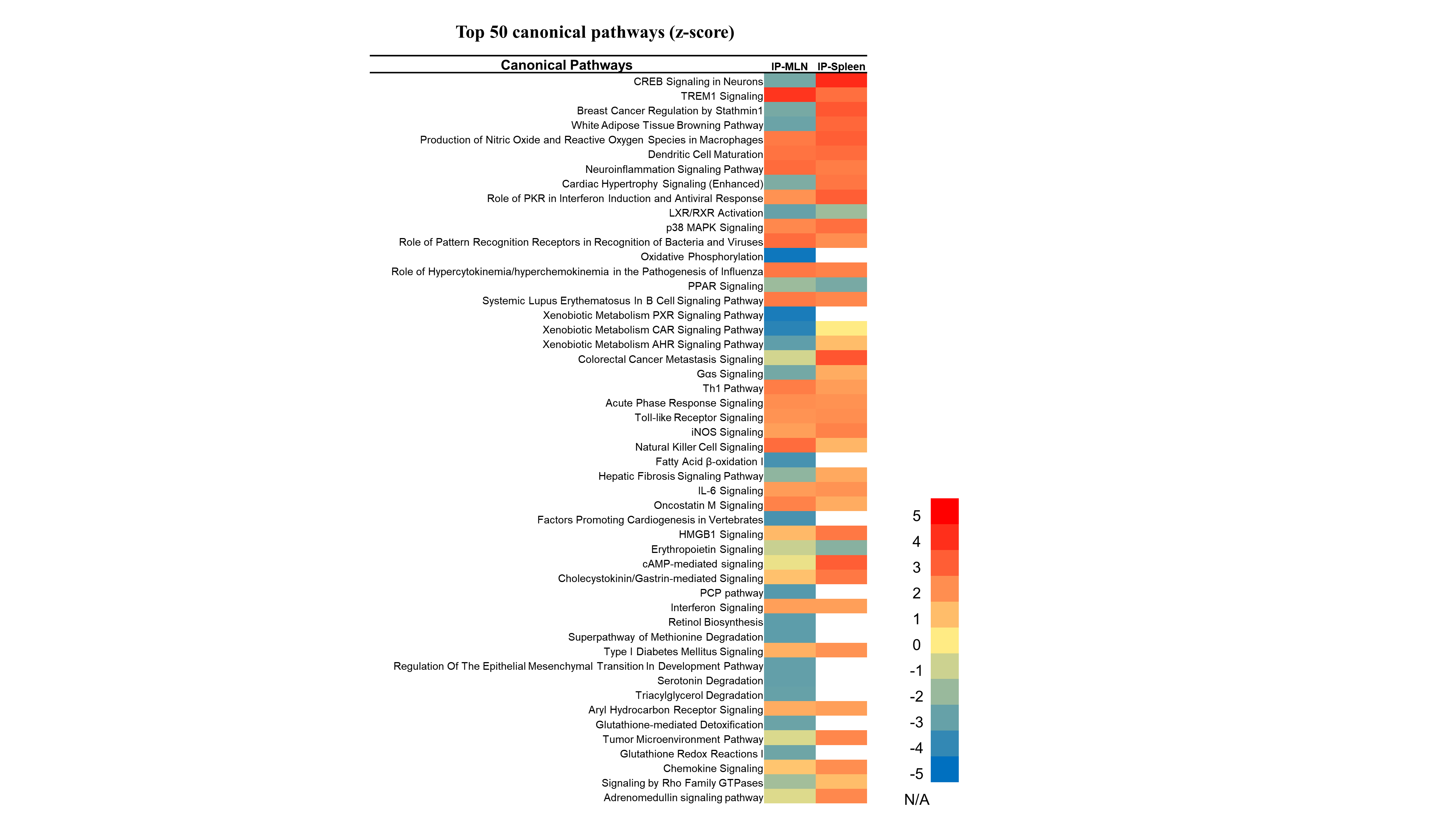

Supplement: S1 Fig — Top 50 canonical pathways sorted by z score from the MLN and spleen in the IP route. Genes that were not significant (p value ≥ 0.05 or Log2FC < 1.0) are shown as N/A. (TIF) [file pone.0281880.s001.tif]

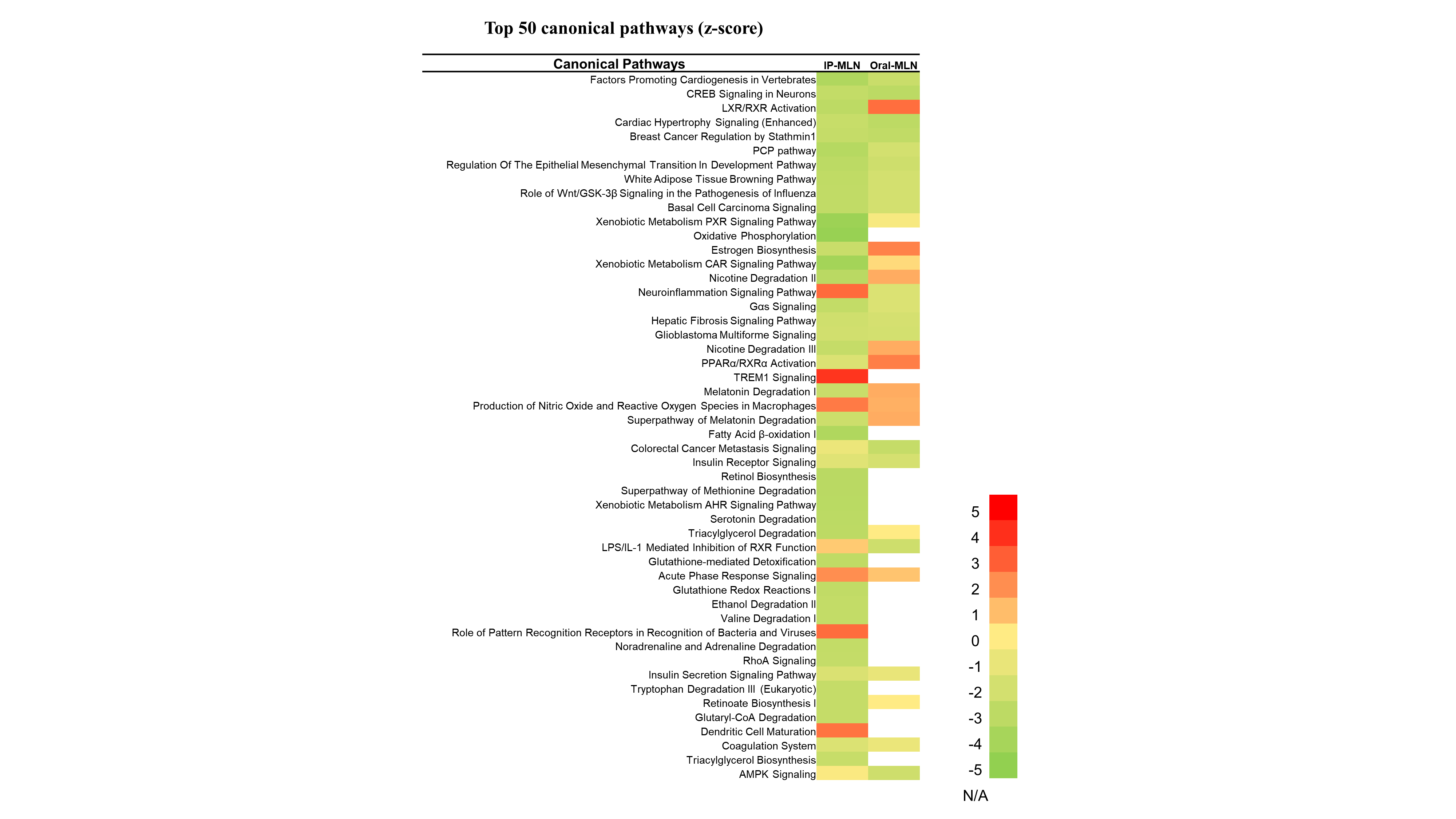

Supplement: S2 Fig — Top 50 canonical pathways sorted by z score in the MLN by the IP and oral routes. Genes that were not significant (p value ≥ 0.05 or Log2FC < 1.0) are shown as N/A. (TIF) [file pone.0281880.s002.tif]

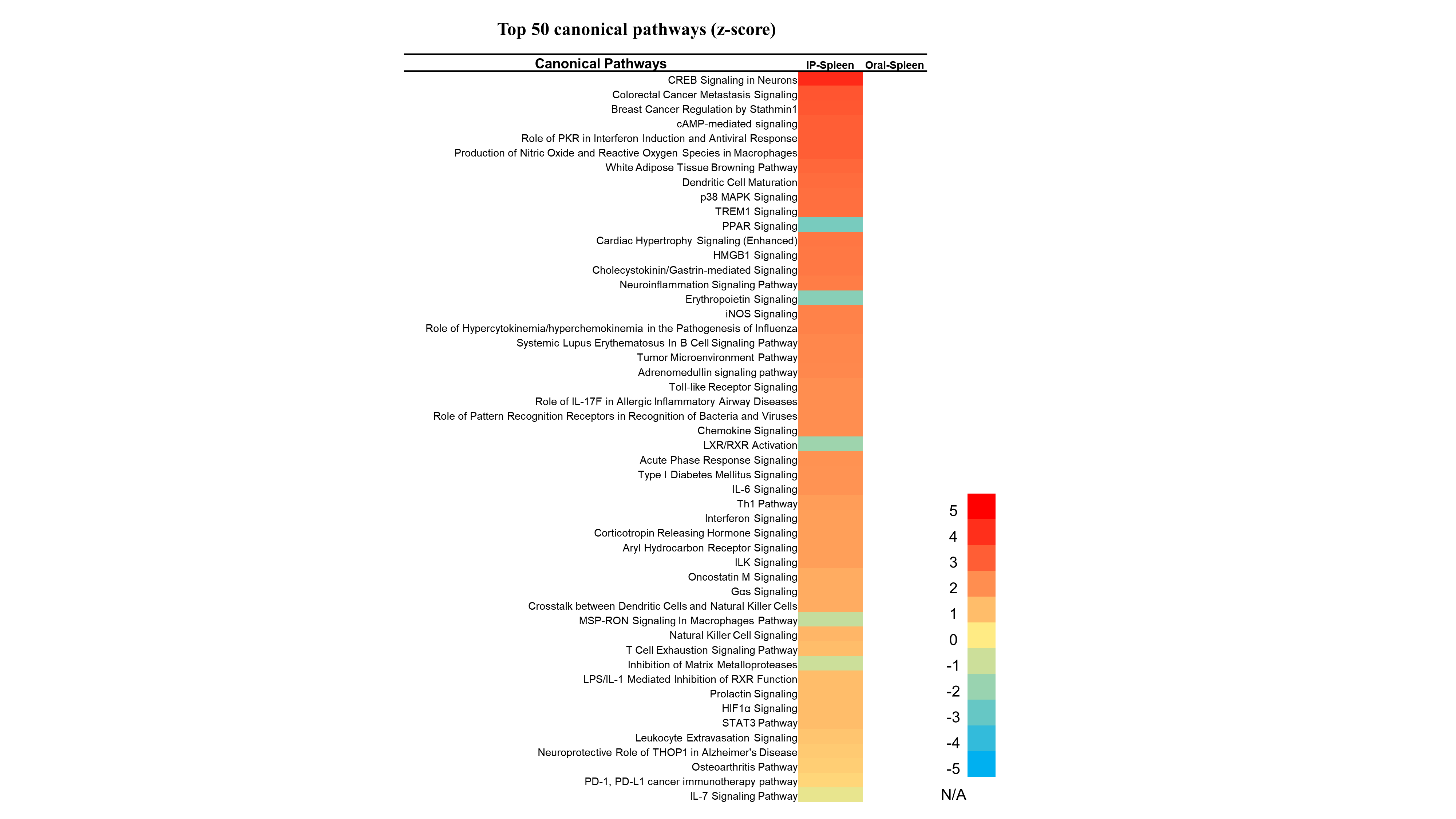

Supplement: S3 Fig — Top 50 canonical pathways sorted by z score in the spleen by the IP and oral routes. Genes that were not significant (p value ≥ 0.05 or Log2FC < 1.0) are shown as N/A. (TIF) [file pone.0281880.s003.tif]
