# Supplementary material for: Immunopathological mechanisms in the early stage of Mycobacterium avium subsp. paratuberculosis infection via different administration routes in a murine model
Source: PLoS One. 2023 Feb 16;18(2):e0281880. doi: 10.1371/journal.pone.0281880 (PMC9934400; doi:10.1371/journal.pone.0281880)
Supplement: S1 Table — (DOCX) [file pone.0281880.s004.docx]

**S1 Table. The 10 most up- or down-regulated genes in the MAP-infected mice (at 6 weeks mouse PI)**

|  | **Gene symbol** | **GeneID** | **Description** | **Fold-change** |
| --- | --- | --- | --- | --- |
| Mesenteric Lymph node – Control/IP | | | | |
| up | *Olfm4* | ENSMUSG00000022026 | olfactomedin 4 | 5.39 |
|  | *Acod1* | ENSMUSG00000022126 | aconitate decarboxylase 1 | 5.25 |
|  | *Ighv9-4* | ENSMUSG00000094322 | immunoglobulin heavy variable 9-4 | 5.20 |
|  | *Gpr84* | ENSMUSG00000063234 | G protein-coupled receptor 84 | 4.91 |
|  | *Gm2808* | ENSMUSG00000116560 | predicted gene 2808 | 4.85 |
|  | *Ms4a3* | ENSMUSG00000024681 | membrane-spanning 4-domains, subfamily A, member 3 | 4.84 |
|  | *4921516A02Rik* | ENSMUSG00000112652 | RIKEN cDNA 4921516A02 gene | 4.82 |
|  | *Gm44775* | ENSMUSG00000108955 | predicted gene 44775 | 4.75 |
|  | *Mcpt8* | ENSMUSG00000022157 | mast cell protease 8 | 4.71 |
|  | *Stfa2l1* | ENSMUSG00000059657 | stefin A2 like 1 | 4.70 |
| down | *Cldn22* | ENSMUSG00000038064 | claudin 22 | -7.81 |
|  | *Amy2a5* | ENSMUSG00000074268 | amylase 2a5 | -6.98 |
|  | *Reg3a* | ENSMUSG00000079516 | regenerating islet-derived 3 alpha | -6.66 |
|  | *Ins2* | ENSMUSG00000000215 | insulin II | -6.41 |
|  | *Gm765* | ENSMUSG00000090667 | MyoD family inhibitor domain containing 2 | -5.96 |
|  | *Gm13610* | ENSMUSG00000086216 | predicted gene 13610 | -5.88 |
|  | *1810010K12Rik* | ENSMUSG00000056329 | RIKEN cDNA 1810010K12 gene | -5.83 |
|  | *Tmed11* | ENSMUSG00000004821 | transmembrane p24 trafficking protein 11 | -5.74 |
|  | *Gm17711* | ENSMUSG00000091367 | predicted gene 17711 | -5.60 |
|  | *Rnu3b2* | ENSMUSG00000105115 |  | -5.51 |
| Mesenteric Lymph node – Control/Oral | | | | |
| up | *Cyp3a11* | ENSMUSG00000056035 | cytochrome P450, family 3, subfamily a, polypeptide 11 | 6.89 |
|  | *Rpl10-ps5* | ENSMUSG00000110679 | ribosomal protein L10, pseudogene 5 | 6.51 |
|  | *Gm14414* | ENSMUSG00000083619 | predicted gene 14414 | 6.50 |
|  | *Apob* | ENSMUSG00000020609 | apolipoprotein B | 6.24 |
|  | *Hpx* | ENSMUSG00000030895 | hemopexin | 6.11 |
|  | *Fgg* | ENSMUSG00000033860 | fibrinogen gamma chain | 5.95 |
|  | *Aldob* | ENSMUSG00000028307 | aldolase B, fructose-bisphosphate | 5.83 |
|  | *Gm37915* | ENSMUSG00000103270 | predicted gene 37915 | 5.75 |
|  | *Gm4799* | ENSMUSG00000071151 | predicted gene 4799 | 5.70 |
|  | *Fgb* | ENSMUSG00000033831 | fibrinogen beta chain | 5.65 |
| down | *Ccl19-ps1* | ENSMUSG00000082902 | chemokine (C-C motif) ligand 19, pseudogene 1 | -5.75 |
|  | *Faim2* | ENSMUSG00000023011 | Fas apoptotic inhibitory molecule 2 | -5.65 |
|  | *Mid1-ps1* | ENSMUSG00000095134 | midline 1, pseudogene 1 | -5.64 |
|  | *Gm2004* | ENSMUSG00000095648 | predicted gene 2004 | -5.25 |
|  | *Rtl3* | ENSMUSG00000047686 | retrotransposon Gag like 3 | -5.09 |
|  | *Igkv3-3* | ENSMUSG00000094478 | immunoglobulin kappa variable 3-3 | -5.02 |
|  | *Capn11* | ENSMUSG00000058626 | calpain 11 | -4.77 |
|  | *Gm45193* | ENSMUSG00000108112 | predicted gene 45193 | -4.55 |
|  | *Gm2274* | ENSMUSG00000098369 |  | -4.50 |
|  | *Dhrs9* | ENSMUSG00000027068 | dehydrogenase/reductase (SDR family) member 9 | -4.50 |
| Spleen – Control/IP | | | | |
| up | *Saa3* | ENSMUSG00000040026 | serum amyloid A 3 | 6.67 |
|  | *Gbp2b* | ENSMUSG00000040264 | guanylate binding protein 2b | 5.74 |
|  | *Gm2436* | ENSMUSG00000112022 | predicted gene 2436 | 5.68 |
|  | *Gm8714* | ENSMUSG00000118051 | predicted gene 8714 | 5.49 |
|  | *Gm50471* | ENSMUSG00000118433 | predicted gene 50471 | 5.39 |
|  | *Pdilt* | ENSMUSG00000030968 | protein disulfide isomerase-like, testis expressed | 5.27 |
|  | *Klhl33* | ENSMUSG00000090799 | kelch-like 33 | 5.17 |
|  | *Apol6* | ENSMUSG00000033576 | apolipoprotein L 6 | 4.86 |
|  | *Gm49392* | ENSMUSG00000114635 | predicted gene 49392 | 4.84 |
|  | *Apol11a* | ENSMUSG00000091650 | apolipoprotein L 11a | 4.82 |
| down | *Derpc* | ENSMUSG00000117748 | DERPC proline and glycine rich nuclear protein | -6.09 |
|  | *Ccl19-ps1* | ENSMUSG00000082902 | chemokine (C-C motif) ligand 19, pseudogene 1 | -5.77 |
|  | *Gm2446* | ENSMUSG00000112690 | predicted gene 2446 | -5.69 |
|  | *Gm13066* | ENSMUSG00000086949 | predicted gene 13066 | -5.10 |
|  | *Mcpt4*  *Gm26920*  *Cpne4*  *Gm36423*  *Gm26879*  *Ighv1-37* | ENSMUSG00000061068  ENSMUSG00000058447  ENSMUSG00000032564  ENSMUSG00000114138  ENSMUSG00000097089  ENSMUSG00000095923 | mast cell protease 4  predicted gene 26920  copine IV  predicted gene 36423  predicted gene 26879  immunoglobulin heavy variable 1-37 | -5.02  -4.49  -4.45  -3.92  -3.90  -3.73 |

| Spleen- Control/Oral | | | | |
| --- | --- | --- | --- | --- |
| up | *Gm50471* | ENSMUSG00000118433 | predicted gene 50471 | 6.05 |
|  | *Gm20708* | ENSMUSG00000093485 | predicted gene 20708 | 5.58 |
|  | *H2ac23* | ENSMUSG00000094248 | H2A clustered histone 23 | 4.98 |
|  | *Gm42417* | ENSMUSG00000109510 | predicted gene 42417 | 4.54 |
|  | *Lenep* | ENSMUSG00000078173 | lens epithelial protein | 4.40 |
|  | *Gm2274* | ENSMUSG00000098369 |  | 4.40 |
|  | *Gm42878* | ENSMUSG00000105340 | predicted gene 42878 | 4.07 |
|  | *Gm49392* | ENSMUSG00000114635 | predicted gene 49392 | 4.07 |
|  | *Ighv1-78* | ENSMUSG00000096326 | immunoglobulin heavy variable 1-78 | 3.90 |
|  | *Gabrb1* | ENSMUSG00000029212 | immunoglobulin heavy variable 1-78 | 3.78 |
| down | *Ccl19-ps1* | ENSMUSG00000082902 | chemokine (C-C motif) ligand 19, pseudogene 1 | -5.85 |
|  | *Rpl15-ps3* | ENSMUSG00000061167 | ribosomal protein L15, pseudogene 3 | -5.54 |
|  | *Gm37206* | ENSMUSG00000103651 | predicted gene 37206 | -4.77 |
|  | *Gm45713* | ENSMUSG00000089989 | predicted gene 45713 | -4.51 |
|  | *Gm45234*  *Krt14*  *Lhx9*  *Igkv4-72*  *Gm27021*  *Hist1h2ap* | ENSMUSG00000107478  ENSMUSG00000045545  ENSMUSG00000019230  ENSMUSG00000076545  ENSMUSG00000097919  ENSMUSG00000094777 | predicted gene 45234  keratin 14  LIM homeobox protein 9  immunoglobulin kappa chain variable 4-72  predicted gene 27021  H2A clustered histone 24 | -4.44  -4.09  -4.09  -4.00  -3.99  -3.99 |
